# The role of FDX1 in granulosa cell of Polycystic ovary syndrome (PCOS)

**DOI:** 10.1186/s12902-021-00775-w

**Published:** 2021-06-15

**Authors:** Zhi Wang, Hui Dong, Li Yang, Ping Yi, Qing Wang, Dongmei Huang

**Affiliations:** 1 Department of Integrated Traditional Chinese and Western Medicine, Tongji hospital, Huazhong University of Science and Technology, Jiefang Road 1095#, 430030 Wuhan, China; 2 Institute of Integrated Traditional Chinese and Western Medicine, Tongji hospital, Huazhong University of Science and Technology, Jiefang Road 1095 #, 430030 Wuhan, China; 3 Department of Rehabilitation Center of Wuhan Puren Hospital, Affiliated Hospital of Wuhan, University of Science and Techn ology, Benxi Street 1#, Qingshan District, 430081 Wuhan, China

**Keywords:** FDX1, PCOS

## Abstract

**Background:**

To explore the development mechanism of PCOS and Transcriptomics was applied to seek the key gene.

**Methods:**

Transcriptomics marked by UID (unique identifier) technique of granulosa cell in PCOS and control women was carried out and key gene was picked up. Then the key gene in granulosa cell was measured by RT-PCR. Two PCOS models modeling with Letrozole and Testosterone Propionate were implemented and the key gene in granulosa cell of ovary was measured by immunohistochemistry to verify the relation with PCOS.

**Results:**

GO-enrich of transcriptomics concentrated in domain steroid metabolism and domain mitochondria. Different genes were sought from coexisting in both domain steroid metabolism and domain mitochondria. Finally, five different genes including CYP11A1、CYB5R1、STAR、FDX1 and AMACR were obtained. RT-PCR was implemented to furtherly verify the downregulating mRNA of FDX1 in PCOS, which showed the consistent outcome with the transcriptomics. Level of FDX1 protein in granulosa cell of antral follicle in two PCOS models was measured and decreased.

**Conclusions:**

FDX1 was related with steroid metabolism and mitochondrial and may participate in the development of PCOS.

## Background

Polycystic ovary syndrome (PCOS) is the most common endocrine disorder in women during their reproductive ages, associated with a plethora of cardiometabolic consequences, with obesity, insulin resistance [1]. As far, the pathogenesis of PCOS was still uncertain. Many mechanisms were mentioned including disturbed HPO axis [2]、hyperinsulinemia[3]、heredity[4] and so on. Granulosa cell was closely neighboring to follicle and played a very import role in the development of follicle, which was paid more attention to the pathogenesis in PCOS. Factors secreted from granulosa cell such as estradiol and insulin-like growth factor were revealed to be essential for follicular development [5]. The granulosa layer is aligned along the follicular basal lamina, no apoptotic cell is observed in healthy follicles [5], but some studies showed that granulosa cell apoptosis increased in PCOS to induce follicular premature atresia [6–9]. For example, DHEA-induced PCOS rat model indicated granulosa cell apoptosis increased [10]. Confusingly, proliferation in granulosa cell of PCOS patients was observed, which was associated with decreased expression of apoptotic effectors and increased expression of a cell survival factor [11]. The status of granulosa cell in PCOS was not elucidated clearly. In this experiment, granulosa cell of PCOS patients and control women was collected and measured by transcriptomics, exploring the abnormal mechanism of granulosa cell in PCOS. Finally, FDX1 was focused owning to relation with mitochondria and ovarian steroidogenesis, which was rarely mentioned in PCOS.

Ferredoxins are low molecular mass proteins that are negatively charged at neutral pH and contain iron-sulfur clusters as a redox active group [12]. Humans possess two mitochondrial ferredoxins, FDX1 and FDX2[13, 14]. FDX1 is essential for the synthesis of various steroid hormones [14]. FDX1 reduces mitochondrial cytochrome P450 enzymes (such as CYP11A1), which catalyze the conversion of cholesterol to pregnenolone, aldosterone, and cortisol [14]. we can see that FDX1 play a very important role in steroid hormones.

Granulosa cell is essential in normal follicular maturation process since they produce steroidal hormones and growth factors[15]. Difference of FDX1 mRNA was found significant between PCOS women and control women after transcriptomics in this study. The relation of FDX1 in granulosa and PCOS was not mentioned. We initially indicated that FDX1 may play a very important role in PCOS.

## Methods

### Participants and Granulosa cell collect

All PCOS women and control women were recruited from Reproductive Medical Center of Tongji Hospital Affiliated to Tongji Medical College, Huazhong University of science and technology. The inclusive criteria of PCOS women are as follow: 1, diagnosed with PCOS; 2, the ages were not more than 34 years old; 3, Except IVF treatment, no other treatments were done in a month. The exclusive criteria of PCOS women; 1, diagnosed with hyperprolactinemia; 2, abnormal androgen secretion due to adrenal or ovarian tumors; 3, patients with uncorrected thyroid disease; 4, suspected Cushing’s syndrome; 5, using estrogen or oral contraceptives and other hormone drugs in recent one month; 6, using other drugs that affect reproductive function or metabolism in the past 2 months (such as anti-obesity drugs, anti-diabetes drugs and traditional Chinese medicine, etc.). Control women receiving IVF treatment were recruited owning to fallopian tube jam、intrauterine adhesions and male factors. The ages of control women were not more than 34 years old. Granulosa cell was collected by Percoll density-gradient centrifugation method[16]. The follicle aspirate was collected and centrifuged at 200 g for 10 min. The supernatant was discarded and the sediment was collected and resuspended with PBS. The resuspended solution was pipetted into the 50 % Percoll gradient (lot number: P4937, sigma) of equal volume and centrifuged at 400 g for 20 min. Intermediate cell layer was collected and resuspended with PBS, which was centrifuged at 200 g for 10 min. Finally, the sediment was collected and deposited at -80℃ refrigerator.

### Total RNA extraction

Total RNA was extracted by Trizol method. 1ml Trizol reagent (Invitrogen, cat. NO 15,596,026) was pipetted into each tube of sediment (granulosa cell) at room temperature for 5 min. After centrifuging at 12,000 g for 15 min, the supernatant was collected into another tube, mixed with 200ul chloroform. After remaining for 15 min at room temperature and centrifuging at 12,000 g 4℃ for 15 min, the water layer of supernatant was removed into a tube mixed with 0.5ml isopropanol, keeping for 10 min at room temperature. After centrifuging at 12,000 g 4℃ for 10 min, the supernatant was discarded and sediment of RNA was collected resuspended with 1 ml 75 % ethanol. After centrifuging at 8000 g 4℃ for 5 min, the supernatant was discarded and sediment of RNA was obtained. The RNA was dissolved with TE buffer and concentration of RNA was measured by Nanodrop™ OneCspectrophotometer (Thermo Fisher Scientific Inc). 1 % agarose gel electrophoresis was administrated to observe the integrity of the strip.

### UID-mRNA-seq

After the total RNA samples were up to standard, 5ug of total RNA was taken for subsequent experiments. KCTM Stranded mRNA Library Prep Kit (Catalog NO. DR08402, Wuhan Seqhealth Co., Ltd. China) was applied in RNA library preparation according to the manufacturer’s instruction. The mRNA was enriched by magnetic beads with oligo (dT). Then, fragment buffer was added to break the resulting mRNA into short fragments. A six base random primer was used to synthesize a single strand of cDNA using the mRNA fragment as a template, and then two-strand cDNA was synthesized by adding buffer, dNTPs and DNA polymerase I. After elution and purification, the terminus of double stranded cDNA was repaired with base and added with sequencing adaptor. The 5 ‘end of cDNA was connected to UID connector. The fragment was caught by magnetic beads and PCR amplification was performed by T100™ Thermal Cycler (BIO-RAD, USA). Agarose electrophoresis was used to detect the quality of the cDNA library. Qubit 3.0 with Qubit™ RNA Broad Range Assay kit (Life Technologies, Q10210) is used to quantify the cDNA library and determine whether the cDNA library concentration is suitable for the computer. After the cDNA library passed the quality inspection, the different cDNA library was sequenced on Illumina sequencer (Illumina NovaSeq 6000) according to the requirements of effective concentration and target offline data volume.

### Data analysis of UID-mRNA-seq

Quality control of raw data included quality scores across all bases and sequence content across all bases. Raw data was filtered via Trimmomatic (version 0.36) and reads of low quality were discarded. Clean data was mapped into the reference genome of human via STAR software (version 2.5.3a). Reads mapped into the exon regions of gene were counted by feature Counts (Subread-1.5.1).

RPKM (Reads per Kilobase per Million Reads) was calculated and normalized to estimate gene expression. Differentially expressed genes were identified using the edgeR package (version 3.12.1) by R4.0.2. Gene expression differences were judged by p-value < 0.05 and fold-change > 2. KEGG enrichment analysis and Go enrichment analysis for differentially expressed genes were administrated by KOBAS software (version 2.1.1).

### Quantitative Real-time PCR

Total RNA was extracted by Trizol method. Reverse transcription PCR was implemented by strand cDNA Synthesis kit (11141ES60, Yeasen, China) according to instruction. Quantitative Real-time PCR was implemented by qPCR kit (11201ES08, Yeasen, China) according to instruction. FDX1, forward primer: CTTTGGTGC ATGTGAGGGAA, reverse primer: GCATCAGCCACTGTTTCAGG. β-actin, forward primer: GTCCACCGCAAATGCTTCTA, reverse primer: TGCTGTCACCT TCACCGTTC. The 2^−ΔΔCT^ method was used for data analysis.

### PCOS modeling

Two PCOS models were implemented. Letrozole and Testosterone Propionate were applied into modeling PCOS. All rats included Wistar weighting 250~300g, purchased from the Hubei Provincial Center for Disease Control and Prevention, Wuhan, China and housed in SPF room of experimental animal center, Tongji Medical College, Huazhong University of science and technology. This animal experiment and all operations were approved by the Institutional Animal Care and Use Committee at Tongji Hospital, Tongji Medical College, Huazhong University of Science and Technology, Wuhan, China. Wistar rats modeling with Letrozole were perfused with Letrozole 1 mg/kg via stomach, continuing for 21days. Control Wistar rats and model Wistar rats were sacrificed at the end of perfusing. Wistar rats modeling with Testosterone Propionate were injected with 10 mg/kg Testosterone Propionate, continuing for 6 weeks. Control rats and model rats injected with Testosterone Propionate were sacrificed at the end of injection. Ovary was picked up and fixed by paraformaldehyde to Paraffin embedding. All rats(n = 6 for each group) were euthanized by intraperitoneal injection with pentobarbital sodium.

### Immunohistochemistry

After paraffin section of ovary dewaxing with xylene and antigen repair with Sodium citrate repair solution, the sections were put into 3 % hydrogen peroxide solution and incubated for 25 min at room temperature. After sections washing for 3 times (each time for 5 min), sections were covered with 10 % goat serum for 30 min. Then, goat serum was discarded and anti-FDX1 antibody (ab108257, Abcam, UK) was added to incubate for the whole night at 4℃. After washing for 3 times (each time for 5 min), sections were incubated with secondary antibody (ab6721, Abcam, UK) for 1 h at room temperature. DAB was added into the section to render color for 1 min. After DAB stain stopping, cell nucleus was stained with Hematoxylin for 1 min. After Hematoxylin stain stopping, sections were added with 1 % alcohol hydrochloride for several seconds and added with Ammonia to turns blue. Finally, the sections were dehydrated with Gradient concentration alcohol and mounted. The length of granulosa cell and quantitative analysis were measured by the software of Image Pro Plus(version 7.0). The unit of length is expressed as pixels.

### Statistics analysis

Student’s t test analysis was applied if data was meeting with normal distribution and homogeneity of variances. Otherwise, Mann-Whitney U was applied. P value < 0.05 was considered significant. IBM SPSS 23 was used to analyze data and GraphPad prism 7 was applied to plot.

## Results

### Characteristics of clinical baseline

21 control women and 22 PCOS women were included in the experiment. Characteristics of clinical baseline were shown in Table 1. Plasma LH and LH/FSH in PCOS women were increased. Plasma PRL in PCOS women was decreased. Plasma AMH in PCOS women was increased.

**Table 1:** Characteristics of clinical baseline. FSH: follicle-stimulating hormone; LH: luteinizing hormone; E_2_: Estradiol; PRL: prolactin; P: progesterone; T: testosterone; AMH: Anti-Mullerian hormone; data was show as Mean ± SD or Median (25 %quantile,75 % quantile)

Characteristics	Control(n = 21)	PCOS(n = 22)	P value
Age	28.52 ± 3.63	28.05 ± 3.11	> 0.05
BMI	20.54 ± 1.77	23.51 ± 4.15	> 0.05
FSH (mIU/ml)	7.62 ± 1.99	6.65 ± 1.53	> 0.05
LH (mIU/ml)	4.14(3.22,5.38)	10.18 ± 6.08	**< 0.05**
LH/FSH	0.62 ± 0.25	1.14(0.86,2.12)	**< 0.05**
E_2_ (pg/ml)	43.12 ± 15.87	44.49(38.68,59.29)	> 0.05
PRL (ng/ml)	16.41 ± 7.04	10.58(8.17,12.70)	**< 0.05**
P(ng/ml)	0.65(0.44,0.80)	0.64 ± 0.41	> 0.05
T(ng/dl)	38.40 ± 16.22	46.36 ± 23.24	> 0.05
AMH (ng/ml)	4.18 ± 1.54	10.51 ± 4.84	**< 0.05**

### UID-mRNA-seq

**Fig. 1:**
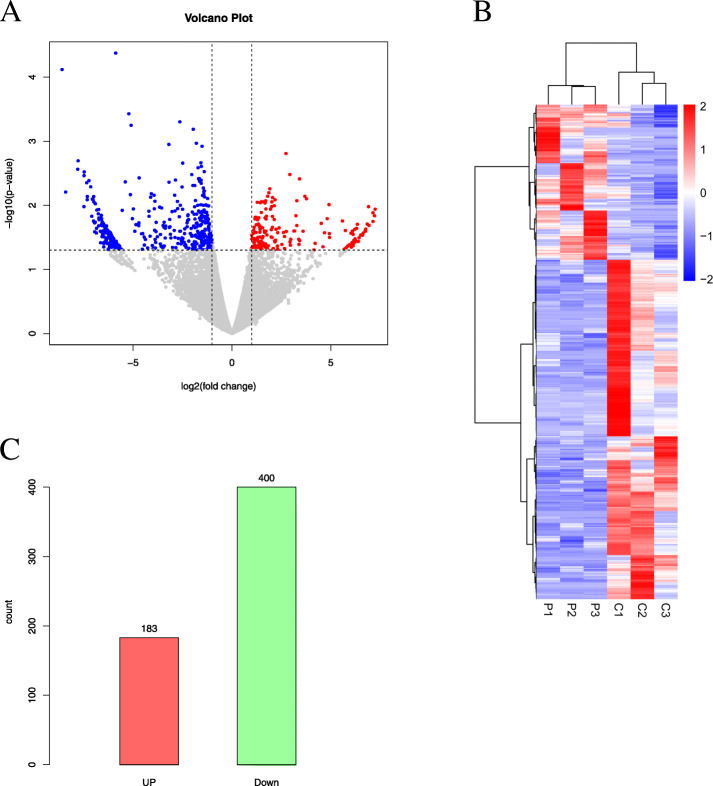
Differentially expressed gene. Volcano plot (**A**)、heatmap plot (**B**) and Bar plot (**C**)

Granulosa cells of 3 PCOS women and 3 control women were measured via UID-mRNA-sEq. Different expression genes were shown in volcano plot (Fig. 1 A) and heatmap plot (Fig. 1B). Totally, 183 up-gene and 400 down-gene in PCOS women were obtained (Fig. 1 C).

### Go enrichment and KEGG enrichment

**Fig. 2:**
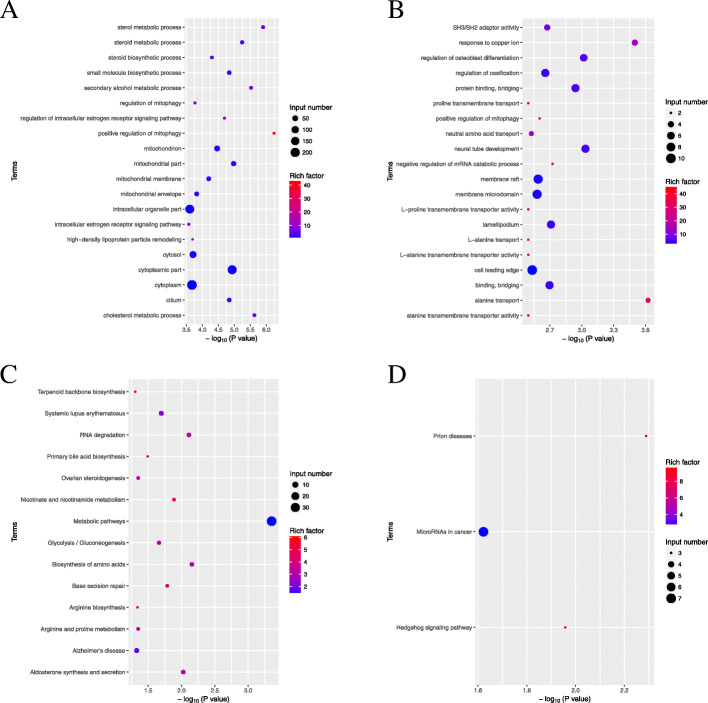
Enrichment analysis including Go enrichment and KEGG enrichment according to data of UID-mRNA-sEq. Go enrichment (**A**: down-gene, **B**:up-gene) and KEGG enrichment (**C**: down-gene, D:up-gene)

Go enrichment and KEGG enrichment according to up-gene and down-gene in PCOS women were implemented. Go enrichment showed that down-gene was mainly located in domain steroid metabolism and domain mitochondria (Fig. 2 A). Go enrichment of up-gene was mainly located in term SH3/SH2 adaptor activity、response to copper ion、regulation of osteoblast differentiation、regulation of ossification and so on (Fig. 2B). KEGG enrichment of down-gene was mainly located in term Terpenoid backbone biosynthesis、Systemic lupus erythematosus、RNA degradation、Primary bile acid biosynthesis、Ovarian steroidogenesis and so on (Fig. 2 C). KEGG enrichment of up-gene was mainly located in term Prion disease、MicroRNAs cancer and Hedgehog signaling pathway (Fig. 2D). Combining Go enrichment and KEGG enrichment, domain steroid metabolism and domain mitochondria of Go enrichment were focused and Ovarian steroidogenesis of KEGG enrichment was focused. Finally, Five genes including CYP11A1、CYB5R1、STAR、FDX1 and AMACR were associated with domain steroid and domain mitochondria. FDX1 was focused in the experiment.

#### FDX1 mRNA expression

**Fig. 3:**
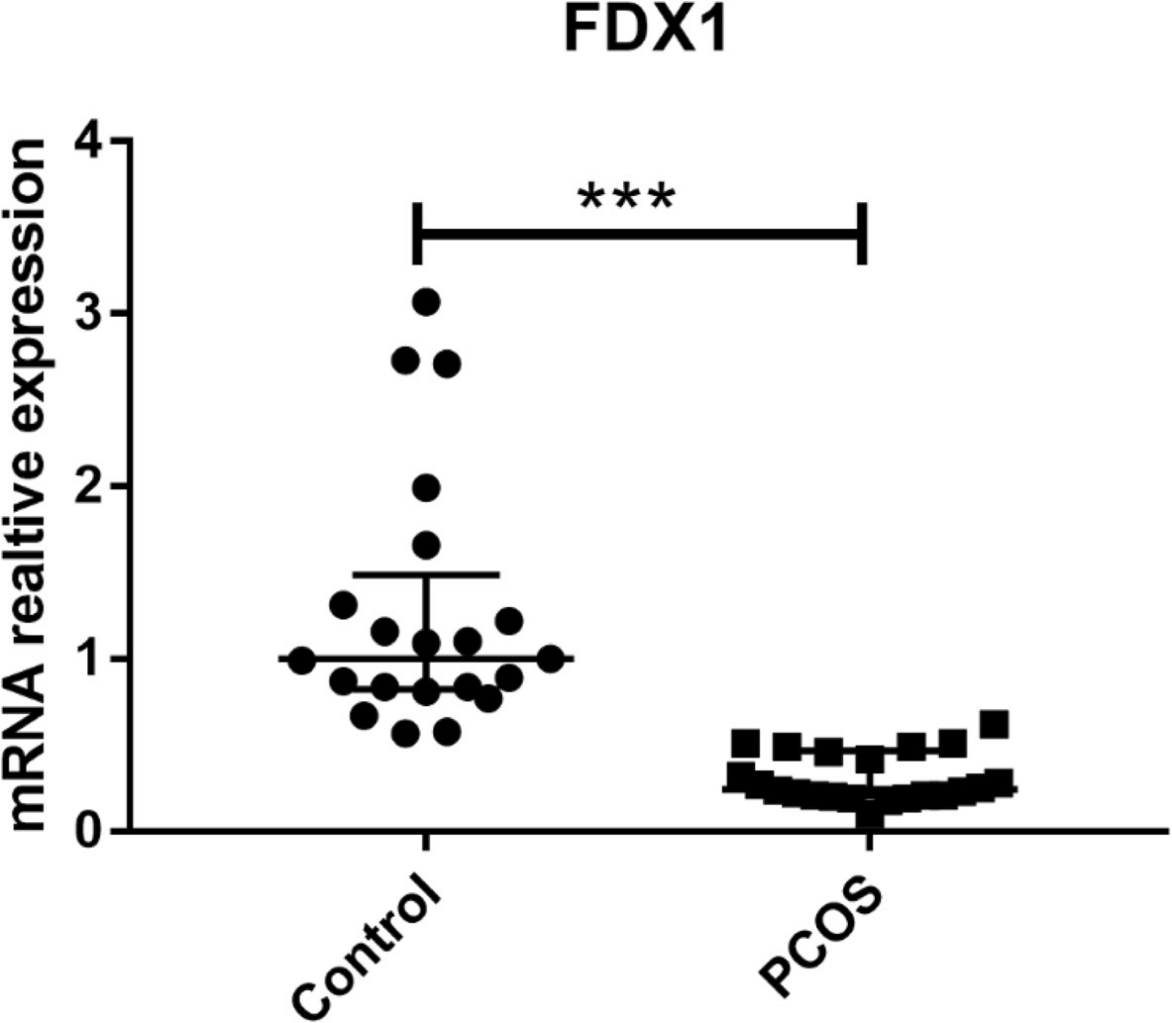
Relative mRNA expression of FDX1 between PCOS women (*n* = 22) and control women (*n* = 21)

To verify the relation between FDX1 and PCOS, mRNA of FDX1 was measured. Granulosa cell of 21 control women and 22 PCOS women were measured by RT-PCR. Expression of FDX1 mRNA in PCOS was decreased.(Fig. 3).

### FDX1 in PCOS model rat

**Fig. 4:**
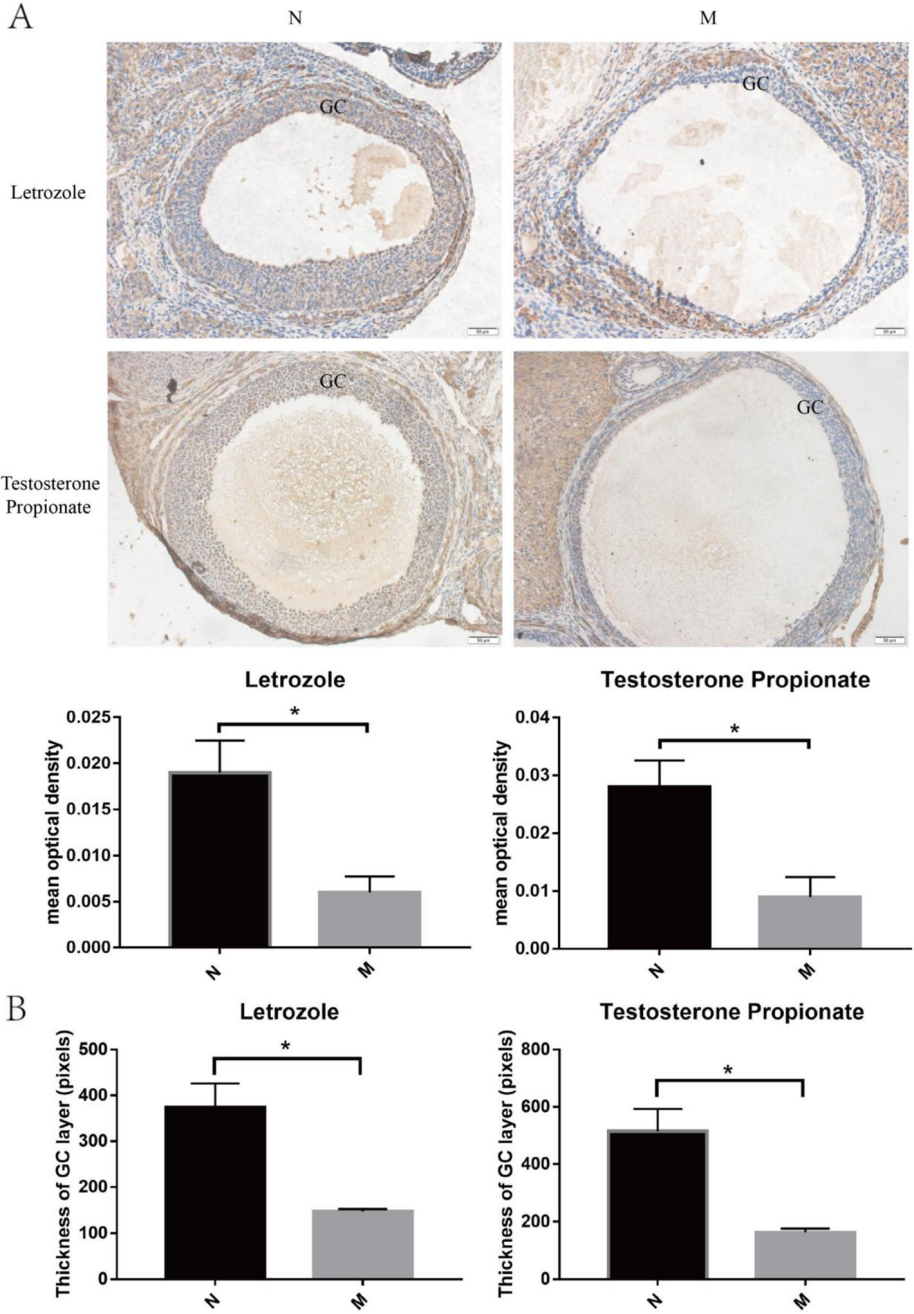
Expression of FDX1 in granulosa cell of antral follicle between normal group and model group by immunohistochemistry. Quantitative analysis was complemented (**A**) and length of GC layer was measured(**B**). Scale bar:50 μm. GC: granulosa cell. N: normal group; M: model group.(*n* = 3 for each group)

Two PCOS models including Letrozole and Testosterone Propionate were used to verify the role of FDX1 in PCOS furtherly. Ovarian morphology of two models was both meeting with the PCOS model establishment. Cystic follicle was obvious in two models and layer of granulosa cell was thinner(Figure 4.B). Protein of FDX1 in PCOS model modeling with Letrozole or Testosterone Propionate was decreased (Fig. 4.A).

.

## Discussion

Profile of transcriptomics of granulosa cell indicated that abnormality of domain steroid and domain mitochondria may be related closely with the development of PCOS. Five down genes including CYP11A1、CYB5R1、STAR、FDX1 and AMACR were associated with domain steroid and domain mitochondria. FDX1 was rarely to be mentioned in PCOS so far. Relative mRNA expression of FDX1 was measured by RT-PCR to verify the transcriptomics, which showed that mRNA of FDX1 in PCOS was decreased. Moreover, two PCOS models modeling with Testosterone Propionate and Letrozole respectively showed that FDX1 protein of granulosa cell of antral follicle in PCOS model was decreased. FDX1 may participate in the development of PCOS and play a very important role in PCOS.

Down-regulating FDX1 had been verified in PCOS, but the mechanism was unclear. FDX1 functions as the electron donor for the catalytic activity of P450 enzyme which catalyzed the conversion of cholesterol to pregnenolone, furtherly pregnenolone converting to progesterone by 3b-hydroxysteroid dehydrogenase (3β-HSD)[17]. Progesterone is an intermediate object in the synthesis of estrogen, androgen and adrenocortical hormone. Moreover, three Cytochrome P450 family including CYP1A1, CYP11A1 and CYP2UL interact with FDX1 to form an interactive network in the ovarian steroidogenesis pathway [18]. Some previous studies showed activity of.

granulosa cell was altered, which caused reduced production of estradiol and progesterone by pre-ovulatory follicle in PCOS[19]. FDX1 was probably linked to the altered activity of granulosa cell. FDX1 in granulosa cell may be closely related with the hormone dysfunction. On the other hand, FDX1 was associated with mitochondria. Mitochondrial dysfunction of human granulosa cells may contribute to the decline of steroidogenesis, decreased fertilization rate, oocyte maturation rate, and oocyte quality, and it can ultimately jeopardize fertility [20]. PCOS model induced by 5α-dihydrotestosterone (DHT) showed that mitochondria tended to fission and apoptosis was observed in granulosa cell at the antral stage of development[9]. Mitochondria fission was closely related with cell apoptosis[21]. Mitochondrial biogenesis genes were downregulated in granulosa cells of PCOS mice when compared to the non-PCOS granulosa cells [22]. So FDX1 probably participated in the development of PCOS.

Furtherly, the status of granulosa cell in pre-antral follicles and antral follicles was different. Increased density of small pre-antral follicles and an increased proportion of early growing follicles were observed in PCOS along with abnormal granulosa cell proliferation[23]. Apoptosis of granulosa cell in antral follicle occurs in PCOS[8]. In this study, FDX1 focused on granulosa cell of antral follicle, leading to the limitation. More work needed to be done in the future, exploring the concrete mechanism of FDX1 in PCOS.

## Conclusions

FDX1 was related with steroid metabolism and mitochondrial and may participate in the development of PCOS.

## Data Availability

All clinical data supporting the conclusions of this article are included within the article. Raw data of transcriptomics could be obtained from SRA database(SRX9530386, SRX9530385, SRX9530384, SRX9530383, SRX9530382, SRX9530381, https://www.ncbi.nlm.nih.gov/sra/PRJNA679416),which was uploaded in NCBI.

## Abbreviations

PCOS: Polycystic ovary syndrome
